# Estimating mean population salt intake in Fiji and Samoa using spot urine samples

**DOI:** 10.1186/s12937-019-0484-9

**Published:** 2019-09-10

**Authors:** Joseph Alvin Santos, Emalie Rosewarne, Martyna Hogendorf, Kathy Trieu, Arti Pillay, Merina Ieremia, Leausa Toleafoa Take Naseri, Isimeli Tukana, Wendy Snowdon, Kristina Petersen, Jacqui Webster

**Affiliations:** 1 The George Institute for Global Health, The University of New South Wales, Sydney, 2052 Australia; 2 Pacific Research Centre for the Prevention of Obesity and Noncommunicable Diseases, Fiji National University, Nasinu, Fiji; 3 Ministry of Health Samoa, Apia, Samoa; 4 National Wellness Centre, Ministry of Health and Medical Services, Suva, Fiji; 5 Global Obesity Centre, Deakin University, Geelong, 3216 Australia; 6 Department of Nutritional Sciences, The Pennsylvania State University, University Park, PA 16802 USA

**Keywords:** Spot urine, 24-h urine, Salt intake, Fiji, Samoa

## Abstract

**Background:**

There is an increasing interest in finding less costly and burdensome alternatives to measuring population-level salt intake than 24-h urine collection, such as spot urine samples. However, little is known about their usefulness in developing countries like Fiji and Samoa. The purpose of this study was to evaluate the capacity of spot urine samples to estimate mean population salt intake in Fiji and Samoa.

**Methods:**

The study involved secondary analyses of urine data from cross-sectional surveys conducted in Fiji and Samoa between 2012 and 2016. Mean salt intake was estimated from spot urine samples using six equations, and compared with the measured salt intake from 24-h urine samples. Differences and agreement between the two methods were examined through paired samples t-test, intraclass correlation coefficient analysis, and Bland-Altman plots and analyses.

**Results:**

A total of 414 participants from Fiji and 725 participants from Samoa were included. Unweighted mean salt intake based on 24-h urine collection was 10.58 g/day (95% CI 9.95 to 11.22) in Fiji and 7.09 g/day (95% CI 6.83 to 7.36) in Samoa. In both samples, the INTERSALT equation with potassium produced the closest salt intake estimate to the 24-h urine (difference of − 0.92 g/day, 95% CI − 1.67 to − 0.18 in the Fiji sample and + 1.53 g/day, 95% CI 1.28 to 1.77 in the Samoa sample). The presence of proportional bias was evident for all equations except for the Kawasaki equation.

**Conclusion:**

These data suggest that additional studies where both 24-h urine and spot urine samples are collected are needed to further assess whether methods based on spot urine samples can be confidently used to estimate mean population salt intake in Fiji and Samoa.

**Electronic supplementary material:**

The online version of this article (10.1186/s12937-019-0484-9) contains supplementary material, which is available to authorized users.

## Background

The World Health Organization (WHO) has recommended that all Member States aim to reduce mean population salt consumption by 30% by 2025, as part of efforts to reduce premature mortality from non-communicable diseases (NCDs) by 25% [1]. Salt reduction has been shown to be one of the most-effective interventions for reducing the burden of NCDs due to its substantial health impact, high feasibility and low implementation costs [2–4]. Even modest reductions in salt intake can lower blood pressure and reduce the risk of cardiovascular diseases (CVDs) [4, 5]. It was projected that a decrease in mean salt intake from 10 to 5 g per day (g/day) would reduce stroke rate by 23% and overall CVD by 17% [6]. This is equivalent to about 1.25 million and 3 million deaths from stroke and CVDs, respectively, averted each year [6].

Accurate measurement of salt intake is essential for setting realistic goals and plans for salt reduction strategies. It is vital to establish baseline salt intake levels and monitor salt consumption regularly to keep track of the population’s progress in reducing salt intake [7]. Currently, 24-h urine collection is the accepted gold standard method for determining daily salt intake in an individual and population [8]. However, the use of 24-h urine collection is limited in some population surveys, since the method is expensive, and can be burdensome to participants due to the complex nature of collection. This often results in poor participation rates and undercollection or overcollection of urine samples [9, 10].

There has been a growing interest in finding less costly and burdensome alternatives to 24-h urine collection, such as spot urine samples. Spot urine collection is done by collecting a small sample of urine from a single void. Studies have shown that while use of spot urine samples is not appropriate for assessing individual-level salt intake, they can provide a reasonable estimate of mean population salt consumption levels [11]. Several equations have been developed to estimate salt intake from spot urine samples. These equations have been tested in different populations; however, most of the studies have focused on developed countries and Western populations [11], and there is little evidence about the usefulness of spot urines in small island developing nations such as Fiji and Samoa. Against this backdrop, this study was conducted to examine whether spot urines can provide a reasonable estimate of population-level salt intake in Fiji and Samoa, with the view to increasing the evidence base in relation to the applicability of spot samples in developing country settings.

## Methods

This study utilized secondary data from the National Health and Medical Research Council (NHMRC)-funded Global Alliance for Chronic Diseases (GACD) project on ‘Cost-effectiveness of salt reduction strategies in the Pacific Islands,’ hereinafter referred to as the GACD Pacific Salt Project. The project was implemented in two countries, Fiji and Samoa, and took place between 2012 and 2016. The study was approved by the University of Sydney’s Human Research Ethics Committee (protocol no: 15359), the WHO Western Pacific Regional Office Ethics and Review Committee, Deakin University (2013–020), the Fiji National Research Ethics and Review Committee (FNRERC 201307), and the Ministry of Health, Samoa Health Research Committee. The study protocol [12] and evaluation of country interventions [13–15] have been published elsewhere.

### The GACD Pacific salt project in Fiji and Samoa: study design and urine collection

In each country, two nationally representative cross-sectional surveys (pre-post design) were conducted to evaluate the change in salt intake after 18–20 months of salt reduction interventions. A multi-stage cluster sampling approach was used to select representative samples of the target population. Briefly, enumeration areas (EA) were selected using probability proportional to size sampling. For each EA, households were randomly selected and within each household, one individual was randomly selected without replacement. Participants were adults between the age of 25 to 64 years in Fiji and 18 to 64 years in Samoa. Participants who were pregnant, lactating or menstruating at the time of the survey were excluded.

Each participant was asked to collect one 24-h urine sample and one spot urine sample anytime during the same 24-h period. Verbal and written instructions on how to accurately collect the samples were given, together with the equipment for collecting urine samples. Participants were instructed to discard the first urine void upon waking in the morning and then collect all their urine for the following 24-h period (24-h urine), and to collect one urine sample in a smaller container (spot urine). Participants recorded the start and finish times of the 24-h urine collection and the time of the spot urine collection. Upon pick-up of the collected samples, participants were asked about any missed voids or spillage. Researchers measured the volume of the urine samples prior to sending to the laboratory for sodium, potassium, and creatinine concentration analysis (potassium concentration was determined at both time points in Samoa, but was only done during the follow-up survey in Fiji).

### Secondary analysis of data from the GACD Pacific Salt Project

#### Participants included in the analyses

These secondary analyses only included participants with complete urine samples (i.e. paired 24-h urine and spot urine samples), and complete data on age, sex, height, weight and body mass index (BMI), since these variables were required to estimate daily salt intake from spot samples using established equations. Given that the completeness of 24-h urine collection could be affected by several factors such as spillage, missed voids, or overcollection (i.e. going beyond the 24-h collection period), the 24-h urine samples were excluded if the total urine volume was < 500 mL, and the total creatinine excretion was < 4 mmol/day or > 25 mmol/day for women, and < 6 mmol/day or > 30 mmol/day for men [15–17]. Spot urines were excluded if the spot creatinine concentration was < 1.8 mmol/L for both sexes, or > 28.3 mmol/L for women and > 32.7 mmol/L for men. These criteria were based on a review of cut-offs employed by various laboratories. Since creatinine excretion is highly variable and can be affected by numerous factors including age, sex, body mass, ethnicity, exercise, and recent diet, among others [10], the widest range was used in this study.

#### Estimation of salt intake from 24-h urine and spot urine samples

All salt intake estimates were reported in g/day. For 24-h urine samples, the 24-h sodium excretion (mmol/day) was obtained by multiplying the sodium concentration (mmol/L) by the urine volume (L). This was then transformed to mg/day by multiplying by 23 (the molar mass of sodium is 23 g/mol) and then by 2.5 (1 mg sodium = 2.5 mg salt). The resulting value was divided by 1000 (1 g = 1000 mg) to obtain salt intake in g/day. For spot urine samples, sodium excretion was estimated using six established equations: Kawasaki [18], Tanaka [19], Mage [20], the International Cooperative Study on Salt, Other Factors, and Blood Pressure (INTERSALT) with or without potassium [21, 22], and Toft [23]. Briefly, the INTERSALT equation is based on a regression model that includes age, sex, BMI, and spot urine measurements (spot sodium, creatinine, with or without potassium) [21, 22]. The other equations estimate 24-h sodium excretion by adjusting the ratio of spot urine sodium and creatinine by the predicted 24-h creatinine excretion. The formulas are shown in Additional file 1.

#### Statistical analysis

In these secondary analyses, data were unweighted since using a weighting scheme would likely inflate the standard errors of the estimates, and lower the statistical power of the comparison between 24-h and spot urine samples. Thus, the salt intake estimates reported in this paper differ from the values previously reported where weighted analyses were conducted to provide nationally representative salt intake estimates based on 24-h urine samples [13–15].

Paired samples t-tests were used to determine the difference in mean salt intake measured from 24-h urine and spot urine samples. Estimates from spot urines were considered as slightly (< 1 g/day), moderately (1 to 2 g/day) or substantially (> 2 g/day) different to 24-h urine samples [24]. A two-way, mixed-effects, single rater, consistency-of-agreement model was used to determine the intraclass correlation coefficient (ICC) of the two methods [25]. Bland-Altman plots were used to determine the agreement between the methods, by plotting the difference in salt intake measured between 24-h urine and spot urine samples on the vertical axis against the mean of the two methods on the horizontal axis [26]. Regression-based lines and the 95% limits of agreement were calculated and added to the Bland-Altman plots to better illustrate the varying limits of agreement due to presence of proportional bias [27]. Finally, the capacity of spot urine samples to classify population-level salt intake as above or below 5 g/day was examined. All these analyses were applied to the country-level datasets. For each country, the baseline and follow-up data points were combined. This was possible since the data were collected from different individuals at each time point and are therefore independent.

Data analyses were carried out using STATA SE V12.0 for Windows (StataCorp LP, Texas). Results are reported as mean, standard deviation (SD), standard error (SE) or 95% confidence interval (CI) where appropriate. All analyses were two-sided and a *p*-value of < 0.05 was used to indicate a significant finding. No missing data were imputed.

## Results

Six hundred sixty nine and 998 individuals in Fiji and Samoa, respectively, provided consent to participate in the surveys. Three participants from Fiji and 89 participants from Samoa were excluded for missing or implausible data. A further 252 participants from Fiji and 184 participants from Samoa were excluded for suspected inaccurate 24-h urine collection (*n* = 233 and 163, respectively) and implausible spot creatinine concentration (*n* = 19 and 21, respectively), leaving a final sample of 414 from Fiji and 725 from Samoa (62% participation rate in Fiji and 73% in Samoa) included in the secondary analyses. It must be noted that for all analyses involving the INTERSALT equation with potassium, the sample size for the Fiji sample was different (*n* = 261) since spot urine potassium concentration was not available during the baseline survey.

The demographic characteristics of the participants included in these secondary analyses are displayed in Table 1. The mean age of the subjects were 45 years in Fiji and 39 years in Samoa, with more women than men in both countries. Approximately half completed primary school or less education in Fiji while about half were educated to secondary level in Samoa. The average BMI was 29 kg/m^2^ in Fiji and 33 kg/m^2^ in Samoa, and mean systolic blood pressure (SBP) and diastolic blood pressure (DBP) were 133 mmHg and 83 mmHg, respectively in Fiji and 130 mmHg and 82 mmHg, respectively in Samoa. Compared to participants from Fiji, participants from Samoa were on average younger, more educated, had higher weight and BMI, and lower SBP (*p* < 0.05). In terms of urine characteristics, participants from Samoa also had lower 24-h urine volume and higher 24-h urine creatinine excretion (p < 0.05).

**Table 1:** Study participant characteristics

Characteristics	Fiji (*n* = 414)		Samoa (*n* = 725)	
Age, years (mean, SD)^a^	45 (11)		39 (13)	
Sex (%)				
Male	45		45	
Female	55		55	
Region (%)	42	Central	16	Apia
	5	Eastern	29	Northwest Upolu
	18	Northern	27	Rest of Upolu
	34	Western	29	Savaii
Education (%)^a^				
Completed primary school or less	48		33	
Completed secondary school	37		51	
Completed tertiary school	15		15	
Height, cm (mean, SD)	167 (10)		167 (8)	
Weight, kg (mean, SD)^a^	81 (19)		92 (21)	
BMI, kg/m^2^ (mean, SD)^a^	29 (6)		33 (7)	
SBP, mmHg (mean, SD)^a^	133 (21)		130 (19)	
DBP, mmHg (mean, SD)	83 (13)		82 (14)	
Urine volume, mL/day (mean, SD)^a^	1684 (742)		1357 (641)	
Creatinine excretion, mmol/day (mean, SD)^a^	10 (5)		13 (5)	

^a^difference between Fiji and Samoa participants significant at *p* < 0.05; *SD* Standard deviation; *BMI* Body mass index, *SBP* Systolic blood pressure, *DBP* Diastolic blood pressure

### Salt intake estimated from 24-h urine and spot urine samples

**Fig. 1:**
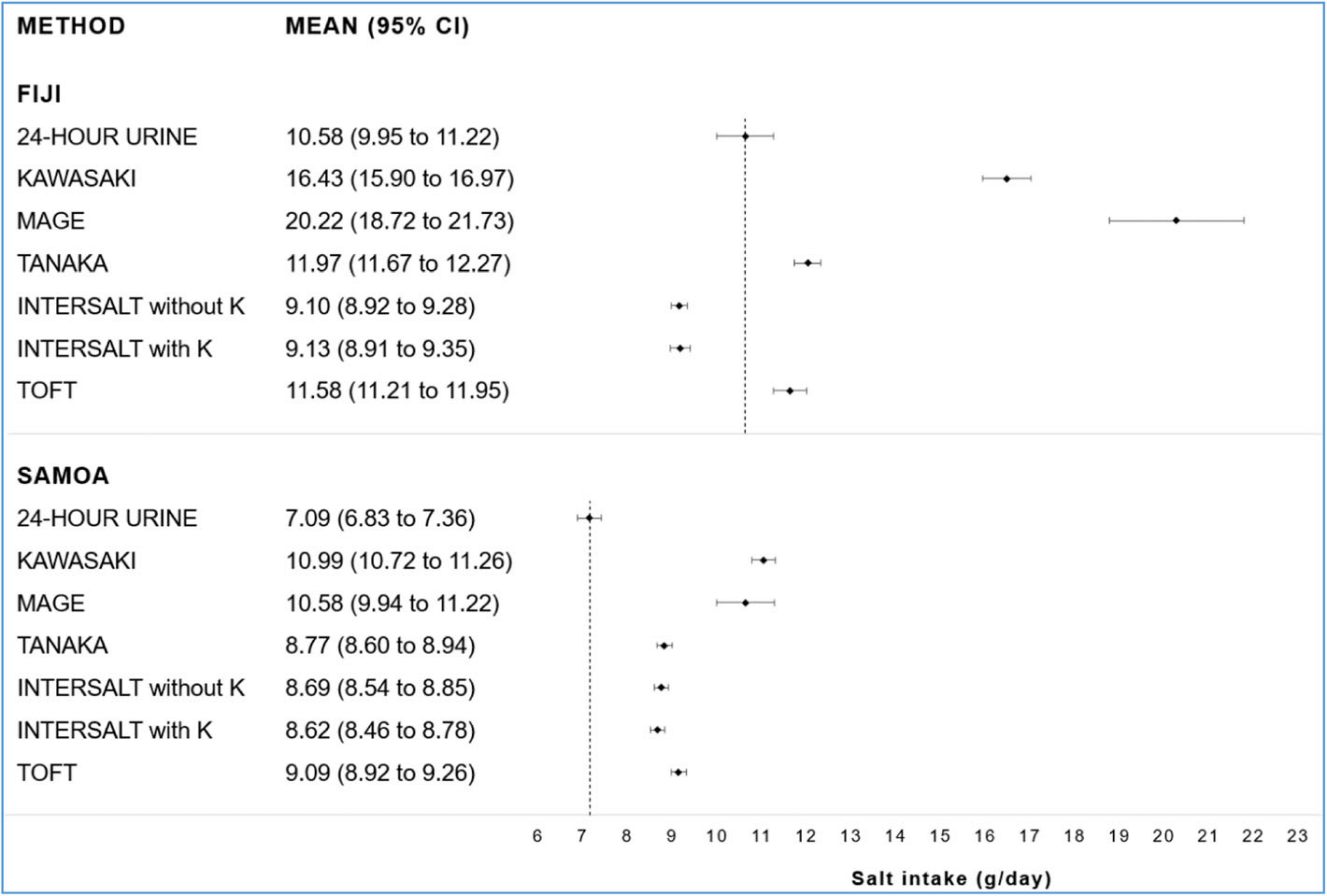
Estimated mean salt intake using 24-h and spot urine samples

Based on the 24-h urine, mean salt intake was 10.58 g/day (95% CI 9.95 to 11.22) in Fiji and 7.09 g/day (95% CI 6.83 to 7.36) in Samoa. The corresponding mean salt intake estimates from spot urine samples using each equation are displayed in Fig. 1. Of the six spot equations, the Mage equation consistently produced the widest CIs. The 24-h urine and all spot equations showed that the population-level salt intake in Fiji and Samoa was above 5 g/day.

### Agreement between 24-h and spot urine samples in assessing salt intake

#### Difference in mean salt intake

All pairwise comparisons of salt intake estimates based on spot urine equations against 24-h urine values were significantly different (all *p*-values < 0.05). The Kawasaki and Mage equation substantially overestimated mean salt intake in both the Fiji (+ 5.85 and + 9.64 g/day) and Samoa sample (+ 3.89 and + 3.49 g/day). Average salt intake was moderately overestimated by the Tanaka and Toft equation in the two samples (Fiji + 1.38 and + 1.00 g/day, and Samoa + 1.68 and + 2.00 g/day). Mean salt intake was moderately overestimated by the INTERSALT equations with and without potassium in the Samoa sample (+ 1.53 and + 1.60 g/day). On the contrary, in the Fiji sample, the INTERSALT equation with potassium slightly underestimated mean salt intake (− 0.92 g/day) while the INTERSALT equation without potassium moderately underestimated salt intake (− 1.48 g/day). These were the only underestimations observed in the study. In both the Fiji and Samoa sample, the INTERSALT equation with potassium produced the closest salt intake estimate to the 24-h urine (− 0.92 and + 1.53 g/day, respectively).

#### Intraclass correlation coefficients

The Mage equation and the two INTERSALT equations had the lowest ICCs, while the Kawasaki equation had the highest in both samples. The spot equations consistently produced lower ICCs in the Fiji sample compared to the Samoa sample (Table 2).

**Table 2:** Intraclass correlation coefficients of each spot equation and 24-h urine

Method	Fiji		Samoa	
	ICC	95% CI	ICC	95% CI
Kawasaki	0.33	0.24 to 0.41	0.50	0.44 to 0.55
Tanaka	0.22	0.12 to 0.31	0.42	0.36 to 0.48
Mage	0.13	0.04 to 0.23	0.29	0.22 to 0.35
INTERSALT without K	0.17	0.08 to 0.26	0.36	0.30 to 0.42
INTERSALT with K	0.13	0.00 to 0.24	0.36	0.30 to 0.42
Toft	0.30	0.21 to 0.39	0.39	0.32 to 0.45

*ICC* Intraclass correlation coefficient, *CI* Confidence interval, *K* Potassium

#### Bland-Altman plots and 95% limits of agreement

**Fig. 2:**
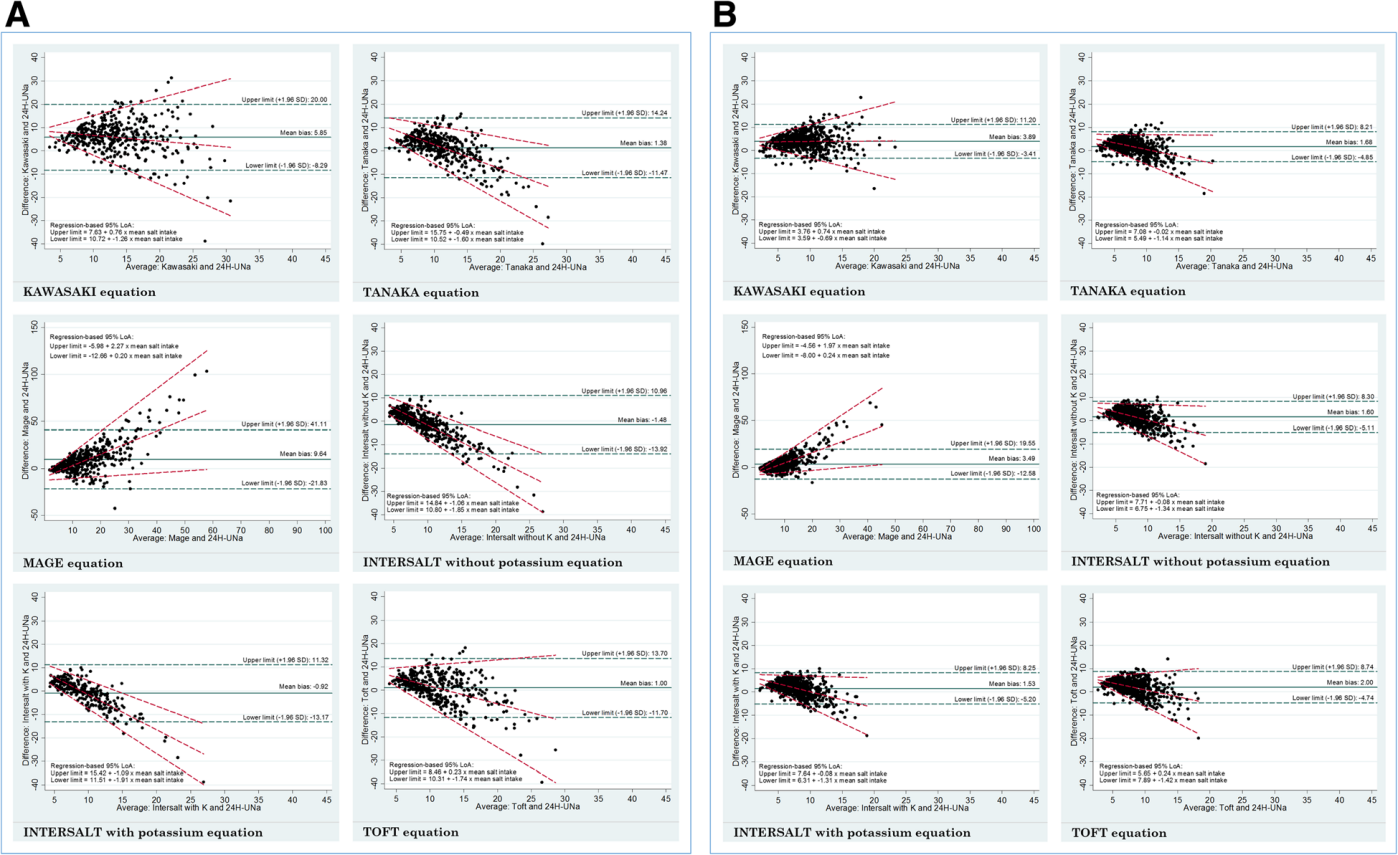
**a**. Bland-Altman plots of salt intake from 24-h urine and each spot equation: Fiji. **b**. Bland-Altman plots of salt intake from 24-h urine and each spot equation: Samoa

Figure 2a and b illustrate the Bland-Altman plots of salt intake estimated from 24-h urine samples and each spot urine equation for each country sample. Apart from the Kawasaki equation, the difference in salt intake between 24-h urine and each spot urine equation was proportional to the level of salt intake. Salt intake estimates from the Tanaka, INTERSALT with and without potassium, and Toft equations were higher at lower levels of intake and lower at higher levels of intake. The opposite was true for the Mage equation where estimates were lower at lower intake levels and higher at higher levels of intake. Furthermore, the regression-based lines showed that the limits of agreement were narrower at lower levels of salt intake and wider at higher levels of intake in both samples.

## Discussion

This study provided an in-depth evaluation of the capacity of spot urine samples to estimate population-level salt intake in Fiji and Samoa. The results indicate substantial heterogeneity in average population-level salt intake estimates from spot urine-based equations in this population. Of the six equations examined, the INTERSALT equation with potassium produced the closest salt intake estimate to the 24-h urine in both samples. This finding is consistent with previous studies conducted in different population groups, including the US [28], Australia [29], Vietnam [15], India [24], New Zealand [30], Ireland [31], and an earlier study in Samoa (2016) that used the GACD Pacific Salt Project data available at that time [32]. The results from all of these studies suggest the considerable applicability of this equation in a range of population groups, despite it being originally developed using data from 29 populations (large sample of more than 5000 participants) in North America and Europe [22]. Nevertheless, although the INTERSALT equation with potassium arguably provided the closest salt intake estimates, it overestimated salt intake in Samoa by about a gram and a half, and underestimated salt intake in Fiji by almost a gram, on average. This opposing result may be due to the different levels of salt intake in the two countries, with Fiji having a higher salt intake (10.6 g/day) than Samoa (7.1 g/day) based on 24-h urine samples. At the individual level, it has been shown that the INTERSALT equation tends to underestimate salt intake at higher levels of consumption and overestimate salt intake at lower levels of consumption [11, 15, 24, 33, 34]. More robust studies where both 24-h urine and spot urine samples are collected are required to fully understand the capacity of spot urine samples to estimate mean salt intake in Fiji and Samoa, before they can be confidently used in these populations.

The Kawasaki and Mage equations generated the highest bias and overestimated salt intake by 55 and 91% respectively in Fiji, and 55 and 49% respectively in Samoa. Previous studies that utilized the Kawasaki equation in different groups reported that it has the tendency to overestimate population salt intake, ranging from 14 to 73% [15, 24, 28–31, 35]. This may be because the Kawasaki equation was modelled using second-morning urine samples, and for most of these studies, this criterion was not applied. In the GACD Pacific Salt Project, the spot urine samples were mostly collected in the afternoon or evening in Fiji, and collected in the morning or early afternoon in Samoa, which may have influenced the performance of this equation. In addition, the Kawasaki equation was developed in a Japanese population with a mean BMI of about 22 kg/m^2^ [18]. The mean BMI in Fiji and Samoa is much higher (29 and 33 kg/m^2^, respectively), which is in line with previous reports that show that the Pacific region has some of the highest rates of overweight and obesity globally [36]. On the other hand, the applicability of the Mage equation appears to vary in different populations, although the direction of bias seems to lean toward overestimation [15, 24, 28–30]. In these secondary analyses, the degree of overestimation was different in the two countries using the Mage equation, which may be owing to the difference in characteristics between the two countries.

Based on 24-h urine samples, mean salt intake in Fiji and Samoa is approximately 6 g and 2 g higher, respectively, than the WHO’s recommended limit. This clearly supports the need for continued efforts to reduce salt intake in these countries. The results showed that all spot urine equations correctly identified mean salt intake as exceeding the 5 g/day limit. This confirms the high sensitivity and specificity of spot urine in classifying mean population salt intake as above 5 g/day reported from a previous meta-analysis [11]. This finding will have implications for using spot urine samples in identifying populations that exceed the recommended daily limit.

In terms of agreement between 24-h and spot urine samples, the ICCs reported in this study were comparable to those described in previous reports [15, 24, 33, 34, 37]. Compared to 24-h urines, all spot urine equations showed poor reliability in estimating daily salt intake [38], adding evidence to the consensus that spot urine sampling is inappropriate for individual-level assessment of salt intake [11]. This may be due to the fact that spot urine sampling involves a single time point and does not take into account the circadian rhythm of urinary sodium excretion. Notably, however, the ICC estimates generated for each spot urine equation in the Fiji sample were lower compared to the corresponding estimates in the Samoa sample. The timing of urine collection might have played a role. In Fiji, almost all samples were collected in the afternoon or evening. A study conducted in 2015 comparing the ICCs of spot urine samples collected at different time points showed that afternoon samples had lower ICCs than morning samples, regardless of the equation used [37]. Furthermore, the ICC analysis in this study used the consistency-of-agreement model, which is more concerned about the degree to which salt intake estimates from spot urines can be equated to the salt intake estimates from 24-h urines, after taking into account some degree of systematic error [38]. The higher ICCs in the Samoa sample show that the salt intake estimates from the spot equations and 24-h urines differed more constantly (by the same value), compared to the Fiji sample. This is important and warrants further investigation as it will have implications for performing mathematic adjustments to estimate salt intake.

Nevertheless, the agreement between 24-h urine and spot urine samples should not be solely based on the ICCs. Correlation analysis has been associated with several limitations. A better method, as stated by many authors [39–42], is assessing the agreement through Bland-Altman plots [26]. In this study, the Bland-Altman plots showed wide limits of agreement between spot and 24-h estimates for individuals for all equations, confirming that spot urine is a poor predictor of 24-h salt intake in individuals. Proportional bias, i.e. under or overestimation according to the level of salt intake, was also evident in the plots and was confirmed by the regression analyses conducted. The presence of proportional bias adds to the complexity of using spot urine samples in the assessment of individual-level salt intake. These findings are similar to previous studies [11, 15, 24, 28, 30, 40, 43, 44]. However, despite these issues, the mean bias line in the plots from the spot urine equations (except for the Kawasaki and Mage equations) was reasonably close to zero (no bias line), suggesting that population-level intake estimates from spot urine were comparable with the 24-h urine samples. This suggests that while methods based on spot urines may be flawed for individual assessment of intake, they might be able to provide estimates of population-level salt intake that are close to the 24-h urine estimates [11, 24]. It is therefore important to continue to collect both 24-h urine and spot urine in adequately large samples, so that the salt intake estimates from spot urine samples (using different equations) can be validated.

This study has a number of strengths and limitations that should be considered when interpreting the results. Strengths include the use of several statistical analyses to assess the agreement between 24-h and spot urine samples that provided strong and convincing evidence that spot urines cannot be utilised for individual assessment of salt intake, and allowed identification of the best equation for estimating mean salt intake in the populations of interest. The baseline and follow-up surveys in Fiji and Samoa included different sets of individuals (unpaired). Countries conducting serial population salt intake surveys often use this type of study design, thus, the issues and challenges in using spot samples identified in this study would be relevant to other settings. Lastly, to the best of our knowledge, this is the first study to apply an exclusion criterion for spot urine samples, based on creatinine concentration. Applying this criteria did not result in many participants being excluded from the analyses (*n* = 19 from Fiji and 21 from Samoa), which might suggest that the criteria to assess 24-h urine completeness is more important than the spot urine criteria. Further studies are needed to investigate this.

Limitations include not validating the completeness of 24-h urine samples using para-aminobenzoic acid (PABA)–the gold standard approach for assessing urine completeness [10]. Furthermore, spot urine samples were collected as part of 24-h urine collections (i.e. not independent), hence, it is possible that the agreements observed in this study were overestimated. Lastly, although the sampling design was intended to recruit participants representative of the population, it is unclear whether the results can be generalized to the target population due to the low response rate from the original surveys in both countries.

## Conclusion

These data suggest that additional studies are needed before spot urine samples can be confidently used to estimate population-level salt intake in Fiji and Samoa. The Kawasaki and Mage equations stood out as poor predictors, while the INTERSALT with potassium equation provided the closest salt intake estimate to the 24-h urine. However, it overestimated salt intake in Samoa by about 1.5 g and underestimated salt intake in Fiji by about 1 g. More studies where both 24-h urine and spot urine samples are collected are required to further evaluate the capacity of spot urines to estimate 24-h salt intake in these populations. Adequate monitoring of salt intake will be vital for carrying out population-based interventions to reduce salt intake, thereby reducing the burden of NCDs.

## Additional files

Additional file 1: Spot equations to estimate 24-h sodium excretion (mmol/day). Contains the six equations used to estimate 24-h sodium excretion from spot urine samples: Kawasaki, Tanaka, Mage, INTERSALT with and without potassium, and Toft. (DOCX 16 kb)

## Data Availability

The datasets analysed for this study are not publicly available; however, are available from the authors upon reasonable request and with permission from the Ministry of Health, Samoa Health Research Committee, and the Pacific Research Centre for the Prevention of Obesity and Non-communicable Diseases in Fiji. The contents of this manuscript are based on a Master’s thesis submitted to the School of Public Health, Faculty of Medicine, The University of Sydney [45]. The initial findings were presented during the 27th Scientific Meeting of the International Society of Hypertension in Beijing, China, in September 2018.

## Abbreviations

BMI: Body mass index
CI: Confidence interval
CVD: Cardiovascular disease
DBP: Diastolic blood pressure
EA: Enumeration areas
g/day: Grams per day
GACD: Global Alliance for Chronic Diseases
ICC: Intraclass correlation coefficient
INTERSALT: International Cooperative Study on Salt, Other Factors and Blood Pressure
NCD: Non-communicable disease
NHMRC: National Health and Medical Research Council
PABA: Para-aminobenzoic acid
SBP: Systolic blood pressure
SD: Standard deviation
SE: Standard error
WHO: World Health Organization
