# Supplementary material for: Estimating mean population salt intake in Fiji and Samoa using spot urine samples
Source: Nutr J. 2019 Sep 10;18:55. doi: 10.1186/s12937-019-0484-9 (PMC6737719; doi:10.1186/s12937-019-0484-9)
Supplement: Supplementary file 1 — Spot equations to estimate 24-h sodium excretion (mmol/day). Contains the six equations used to estimate 24-h sodium excretion from spot urine samples: Kawasaki, Tanaka, Mage, INTERSALT with and without potassium, and Toft. (DOCX 16 kb) [file 12937_2019_484_MOESM1_ESM.docx]

**Additional file 1. Spot equations to estimate 24-hour sodium excretion (mmol/day)**

| **Kawasaki equation, 1993** | |
| --- | --- |
| **M** | ${\text{16.3 x }\left\{ \left[ \frac{\text{SNa }}{\text{SCr x 10}} \right]\text{ x }\left[ \left( \text{-12.63 x age} \right)\text{ + }\left( \text{15.12 x wt} \right)\text{ + }\left( \text{7.39 x ht} \right) \text{- 79.9} \right] \right\}}^{\text{0.5}}$ |
| **F** | ${\text{16.3 x} \left\{ \left[ \frac{\text{SNa}}{\text{SCr} \text{ x 10}} \right]\text{ x} \left[ \left( \text{-4.72 x age} \right)\text{ + }\left( \text{8.58 x wt} \right)\text{ + }\left( \text{5.09 x ht} \right) \text{- 74.5} \right] \right\}}^{\text{0.5}}$ |
| **Tanaka equation, 2002** | |
|  | ${\text{21.98 x }\left\{ \left[ \frac{\text{SNa }}{\text{SCr x 10}} \right]\text{ x }\left[ \left( \text{-2.04 x age} \right)\text{ + }\left( \text{14.89 x wt} \right)\text{ + }\left( \text{16.14 x ht} \right) \text{- 2244.45} \right] \right\}}^{\text{0.392}}$ |
| **Mage equation, 2008** | |
| **M** | $\left[ \frac{\text{SNa}}{\text{SCr x 10}} \right]\text{ x} \left[ \text{0.00179 x} \left( \text{140 - age} \right)\text{ x} \left( \text{wt}^{\text{1.5}}\text{ x} \text{ht}^{\text{0.5}} \right) \right]\text{ x} \left[ \text{1 + 0.18 x A x} \left( \text{1.366 - 0.0159 x BMI} \right) \right]$  where A = 1 if African American or Black race, and A = 0 if another race |
| **F** | $\left[ \frac{\text{SNa}}{\text{SCr x 10}} \right]\text{ x} \left[ \text{0.00163 x} \left( \text{140 - age} \right)\text{ x} \left( \text{wt}^{\text{1.5}}\text{ x} \text{ht}^{\text{0.5}} \right) \right]\text{ x} \left[ \text{1 + 0.18 x A x} \left( \text{1.429 - 0.0198 x BMI} \right) \right]$  where A = 1 if African American or Black race, and A = 0 if another race |
| **INTERSALT with potassium equation (North America), 2013** | |
| **M** | $\left\{ \text{25.46 + }\left[ \text{0.46 x SNa} \right] \text{- }\left[ \text{2.75 x SCr} \right] \text{- }\left[ \text{0.13 x SK} \right]\text{ + }\left[ \text{4.10 x BMI} \right]\text{ + }\left[ \text{0.26 x age} \right] \right\}$ |
| **F** | $\left\{ \text{5.07 + }\left[ \text{0.34 x SNa} \right] \text{- }\left[ \text{2.16 x SCr} \right] \text{- }\left[ \text{0.09 x SK} \right]\text{ + }\left[ \text{2.39 x BMI} \right]\text{ + }\left[ \text{2.35 x age} \right] \text{- }\left[ \text{0.03 x }\text{age}^{\text{2}} \right] \right\}$ |
| **INTERSALT without potassium equation (North America), 2013** | |
| **M** | $\left\{ \text{23.51 + }\left[ \text{0.45 x SNa} \right] \text{- }\left[ \text{3.09 x SCr} \right]\text{ + }\left[ \text{4.16 x BMI} \right]\text{ + }\left[ \text{0.22 x age} \right] \right\}$ |
| **F** | $\left\{ \text{3.74 + }\left[ \text{0.33 x SNa} \right] \text{- }\left[ \text{2.44 x SCr} \right]\text{ + }\left[ \text{2.42 x BMI} \right]\text{ + }\left[ \text{2.34 x age} \right] \text{- }\left[ \text{0.03 x }\text{age}^{\text{2}} \right] \right\}$ |
| **Toft equation, 2014** | |
| **M** | ${\text{33.56 x }\left\{ \left[ \frac{\text{SNa }}{\text{SCr x 10}} \right]\text{ x }\left[ \left( \text{-7.54 x age} \right)\text{ + }\left( \text{14.15 x wt} \right)\text{ + }\left( \text{3.48 x ht} \right) \text{+ 423.15} \right] \right\}}^{\text{0.345}}$ |
| **F** | ${\text{52.65 x }\left\{ \left[ \frac{\text{SNa }}{\text{SCr x 10}} \right]\text{ x }\left[ \left( \text{-6.13 x age} \right)\text{ + }\left( \text{9.97 x wt} \right)\text{ + }\left( \text{2.45 x ht} \right) \text{+ 342.73} \right] \right\}}^{\text{0.196}}$ |

SNa = spot sodium; SCr = spot creatinine; SK = spot potassium; ht = height; wt = weight; BMI = body mass index; SNa in mmol/L; SK in mmol/L; SCr in mmol/L for the INTERSALT equations, in mg/dL for the other four equations; age in years; weights in kilograms; height in centimetres; BMI in kg/m^2^
